# An Open Randomized Comparison of Gatifloxacin versus Cefixime for the Treatment of Uncomplicated Enteric Fever

**DOI:** 10.1371/journal.pone.0000542

**Published:** 2007-06-27

**Authors:** Anil Pandit, Amit Arjyal, Jeremy N. Day, Buddhi Paudyal, Sabina Dangol, Mark D. Zimmerman, Bharat Yadav, Kasia Stepniewska, James I. Campbell, Christiane Dolecek, Jeremy J. Farrar, Buddha Basnyat

**Affiliations:** 1 Patan Hospital, Lagankhel, Lalitpur, Nepal; 2 Oxford University Clinical Research Unit, Hospital for Tropical Diseases, Ho Chi Minh City, Vietnam; 3 Centre for Tropical Medicine, Nuffield Department of Clinical Medicine, Oxford University, Oxford, United Kingdom; 4 Regional Infectious Diseases Unit, North Manchester General Hospital, Manchester, United Kingdom; 5 Nepal International Clinic, Kathmandu, Nepal; Liverpool School of Tropical Medicine, United Kingdom

## Abstract

**Objective:**

To assess the efficacy of gatifloxacin versus cefixime in the treatment of uncomplicated culture positive enteric fever.

**Design:**

A randomized, open-label, active control trial with two parallel arms.

**Setting:**

Emergency Room and Outpatient Clinics in Patan Hospital, Lagankhel, Lalitpur, Nepal.

**Participants:**

Patients with clinically diagnosed uncomplicated enteric fever meeting the inclusion criteria.

**Interventions:**

Patients were allocated to receive one of two drugs, Gatifloxacin or Cefixime. The dosages used were Gatifloxacin 10 mg/kg, given once daily for 7 days, or Cefixime 20 mg/kg/day given in two divided doses for 7 days.

**Outcome Measures:**

The primary outcome measure was fever clearance time. The secondary outcome measure was overall treatment failure (acute treatment failure and relapse).

**Results:**

Randomization was carried out in 390 patients before enrollment was suspended on the advice of the independent data safety monitoring board due to significant differences in both primary and secondary outcome measures in the two arms and the attainment of a priori defined endpoints. Median (95% confidence interval) fever clearance times were 92 hours (84–114 hours) for gatifloxacin recipients and 138 hours (105–164 hours) for cefixime-treated patients (Hazard Ratio[95%CI] = 2.171 [1.545–3.051], p<0.0001). 19 out of 70 (27%) patients who completed the 7 day trial had acute clinical failure in the cefixime group as compared to 1 out of 88 patients (1%) in gatifloxacin group(Odds Ratio [95%CI] = 0.031 [0.004 – 0.237], p<0.001). Overall treatment failure patients (relapsed patients plus acute treatment failure patients plus death) numbered 29. They were determined to be (95% confidence interval) 37.6 % (27.14%–50.2%) in the cefixime group and 3.5% (2.2%–11.5%) in the gatifloxacin group (HR[95%CI] = 0.084 [0.025–0.280], p<0.0001). There was one death in the cefixime group.

**Conclusions:**

Based on this study, gatifloxacin is a better treatment for uncomplicated enteric fever as compared to cefixime.

**Trial Registration:**

Current Controlled Trials ISRCTN75784880

## Introduction

Enteric fever (Typhoid and Paratyphoid fever) is a systemic infection caused by the bacterium *Salmonella enterica* serovar Typhi (S.typhi) or *Salmonella enterica* serovar Paratyphi (S. paratyphi) which in humans is transmitted through the fecal-oral route [1], [2]. Today the vast burden of disease is encountered in the developing world where sanitary conditions remain poor. The best global estimates are of at least 22 million cases of typhoid fever each year with 200,000 deaths [3]. Crucially these are almost exclusively confined to resource poor countries. A recent Cochrane review [4] on typhoid treatments underscored the need for large sample size drug interventional trials, especially in children in whom this disease predominates.

In 1948 the introduction of chloramphenicol revolutionized the treatment of typhoid fever [5], [6]. Unfortunately the emergence of resistance to the “first line” antimicrobials (for example, ciprofloxacin) has been a major setback and has given rise to the possibility of untreatable enteric fever [7], [8]. Gatifloxacin, a relatively inexpensive fluoroquinolone antibiotic in South Asia with once daily oral administration, is a new broad spectrum synthetic 8-methoxyfluoroquinolone which has the lowest minimum inhibitory concentration (MIC) against S. typhi from Nepal [9]. This in vitro activity needs to be verified clinically before gatifloxacin can be recommended for widespread use. Cefixime, an orally administered third generation cephalosporin, is a commonly used drug in South Asia for the treatment of enteric fever. Although cefixime is recommended as a drug of choice by the World Health Organization (WHO) for the treatment of resistant typhoid fever [10] it is relatively expensive in South Asia and has to be administered for a longer duration than the currently used fluoroquinolones. Clearly there is an urgent need for a treatment [11] that combines ease of oral administration, with speed of clinical response, reduction in secondary transmission and inexpensiveness. In this open randomized trial, we aimed to compare clinical outcomes for the treatment of uncomplicated enteric fever with gatifloxacin or cefixime in an outpatient setting.

## Methods

### Participants

The study was approved by Nepal Health Research Council and Oxford Tropical Research Ethics Committee. The protocol for this trial and supporting CONSORT checklist are available as supporting information; see Checklist S1 and Protocol S1. We enrolled patients who presented to the outpatient or emergency department of Patan Hospital, Lalitpur, Nepal from June 5, 2005 to September 8, 2005. Patan Hospital is a 318 –bed hospital located in the Lalitpur district in Kathmandu Valley. Patients were eligible to enter the study if they had clinically diagnosed enteric fever and their residence was within approximately 2.5 km radius from the hospital. Other inclusion criteria were that patients must be aged between 2 and 65 years, able to take oral medications, non-pregnant and non-lactating, without a history of seizures, able to stay in the city for the duration of the treatment, not known to have contraindications to either cephalosporins or fluoroquinolones and willing to give informed written consent to take part in the study. For children enrolled into the study, written informed consent was taken from a parent. Patients were excluded from the study if they had any signs of complicated typhoid defined as the presence of jaundice, gastrointestinal bleeding, peritonism, shock, encephalopathy, convulsions, myocarditis or arrythmia at the time of enrollment. Patients who had received a third generation cephalosporin, fluoroquinolone or macrolide in the week prior to presentation to our clinic were also excluded.

### Interventions

On presentation to Patan Hospital all patients with fever without an obvious focus were referred to the enteric fever study clinic, where they were seen by the study physician. Patients who fulfilled the inclusion criteria were randomly assigned to receive Gatifloxacin (Broadband™, Novartis AG Basel, Switzerland) 10 mg/kg/day [12], in a single dose orally for 7 days or Cefixime (Cifex™, Aegis, Nicosia, Cyprus) 20 mg/kg/day [13] in two divided doses orally for 7 days. Both drugs were administered in tablet form, cut and weighed in a sensitive scale to ensure that underdosing did not occur. To children who were apprehensive of swallowing the tablet, the drug was embedded in a banana and given. All patients were asked to swallow the study drug under direct observation during each visit.

Each patient had haematocrit, total leucocyte count with differential, serum creatinine, total bilirubin, alanine aminotranferase(ALT), and aspartate aminotransferase(AST) measured, and blood and stool cultures were also performed before the start of the study intervention.

The exact location of the patient's home was recorded and the first dose of drug administered at the clinic. We employed six Community Medical Auxiliaries (CMA) who had all received at least 15 months of prior formal primary health care worker training and been registered in a government recognized institution. The CMAs visited patients twice daily at their homes to perform a simple clinical assessment, measure the oral temperature and give directly observed therapy with the study drugs. The CMA visited the patient's home every 12 hours, morning and evening, until day 10 following enrollment or complete resolution of illness, whichever came later. The oral temperature of the patient was recorded twice every day by the CMA and a note was made of the timing and dosages of acetaminophen intake. The quality of patient-visits was ensured by regular unplanned supervisory checks in which the study doctor accompanied the CMA during the visits to patients' homes

CMAs were asked to send patients immediately to the hospital on encountering any severe symptoms, and the patients also were asked to attend clinic if they had any severe symptoms at any other time. A symptom questionnaire was used daily during each visit to monitor any adverse events. Any patient with any severe symptom was seen by the study physician. The CMAs and study physicians held daily case conferences at which all the study patients were discussed.

All patients regardless of the culture results were seen at hospital on Day 10 following enrollment. Blood and stool cultures were repeated on Day 10 in all culture positive patients and thereafter if the patient again became ill with probable enteric fever. All culture positive patients were followed up until six months after enrollment, and stool cultures were performed at the end of the first, third and sixth month.

#### Microbiological Procedures

Blood culture was performed on media containing tryptone soya broth and sodium polyethanol sulphonate, incubated at 37 C and examined daily for growth over 7 days [11]. *Salmonella enterica* serotype Typhi or Paratyphi A, B or C isolated in culture were identified using standard biochemical tests and specific antisera (Murex Biotech, Dartford, England). Antibiotic susceptibilities were determined during isolation using the Kirby-Bauer disc diffusion method involving antibiotic discs containing Nalidixic acid, Ofloxacin, Ciprofloxacin, Chloramphenicol, Ampicillin, Cotrimoxazole, Cefixime and Cefotaxime (HiMedia Laboratories, Mumbai, India). Minimum Inhibitory Concentrations (MICs) were determined later for organisms stored in glycerol (bacterial preserver) at -70C. The MICs were determined by Chloramphenicol, Nalidixic acid, Gatifloxacin, Cefixime, Ceftriaxone and Gemifloxacin E-tests™ (AB Biodisk, Solna, Sweden), according to the manufacturer's instructions. The sensitivity tests were interpreted using Clinical and Laboratory Standards Institute criteria for Enterobacteriaceae.

### Objectives

The objective of the study was to compare the efficacy of Gatifloxacin and Cefixime in the treatment of uncomplicated culture positive enteric fever.

### Outcomes

The primary outcome was the fever clearance time (FCT). FCT was defined as time to first drop in oral temperature ≤ 37.5°C, remaining ≤ 37.5°C for 48 hours. The secondary outcomes included acute treatment failure. Acute treatment failure was defined as including any severe complication; the persistence of fever (> 38 C); the persistence of symptoms for more than 7 days after the start of treatment , requiring additional or rescue treatment. If a patient had a temperature above 37.5 and below 38 for more than 7 days, but did not need additional or rescue therapy, and subsequently their fever cleared by day 10, that patient would not qualify as an acute treatment failure. Patients who failed the study treatment were given rescue treatment.

The rescue drug was Ofloxacin 20 mg/kg/day orally in two divided doses for 14 days for the Cefixime group, and Ceftriaxone 40 mg/kg/day IV in a single daily dose for 14 days for the Gatifloxacin group. For the Cefixime group alone, if on day 8 of treatment the patient still had a fever of > = 38°C, the study drug was continued for 10 days and the patient categorized as acute treatment failure. If the temperature on Day 10 was >37.5, rescue treatment was given.

A relapse was defined as fever with a positive blood culture within a month of completing treatment. All the relapses were patients that were initially categorized as successfully treated. Any patient given rescue treatment or prolonged treatment was precluded from the “relapse” group.

Patients categorized as “overall treatment failures” included patients experiencing acute treatment failure, plus those falling into the relapsed category, plus all deaths within the trial follow up period.

### Sample size

The sample size was calculated to detect a FCT difference of approximately 48 hours between gatifloxacin (assumed median FCT 156 hrs) and cefixime (assumed median FCT 204 hrs) [14] with p = 0.05 and power = 80%. The accrual time for recruitment was assumed to last 70 days, and that the last patient would be followed up until 8 days after recruitment. Therefore, we estimated the minimum sample size at 235 participants. Assuming a loss to follow-up of 5%, the sample size was calculated as 125 blood culture positive patients in each arm.

Before the recommended sample size had been reached, once 169 blood culture positive patients had been enrolled, the independent data safety monitoring committee (DSMC) advised the Principal Investigators to stop recruitment to the trial based on a priori defined difference (p<0.01) between the two treatment arms in the primary endpoints of the study.

### Randomization—Sequence generation

Patients were randomized in blocks of 100 from a computer generated randomization list, by an investigator not involved in patient recruitment or assessment.

### Randomization—Allocation concealment

The randomization sequence and block size was concealed from the physicians allocating treatment and managing the patients, prior to patient enrollment. Treatment allocations were kept in sealed opaque envelopes, which were opened only on enrollment of the patient to the study after all inclusion and exclusion criteria had been checked.

### Randomization—Implementation

Participants were enrolled by the study physician in the same order in which they presented to the study clinic. The sealed envelopes were opened in strict numeric sequence.

### Blinding

Blinding was not feasible in this trial due to logistical reasons.

### Statistical methods

All data were entered into an electronic database (Microsoft Office Access Version 2003, Wash., USA), and analyses was performed using Stata 9 (Stats Corp LP; Texas, USA). ). Continuous covariates were compared between groups of patients using the Mann-Whitney test, and categorical covariates were compared using the chi-square test or Fisher's exact test when appropriate. Fever clearance times and time to relapse were analyzed using Kaplan Meier survival curves and compared between the two groups using the logrank test. Binary outcomes (clinical failures) were compared between the two treatment groups using Fisher's exact test. Analysis was done in all randomized patients (intention to treat, ITT) and separately in patients with positive pretreatment culture (per protocol, PP) and negative pretreatment culture.

## Results

### Participant flow

**Figure 1:**
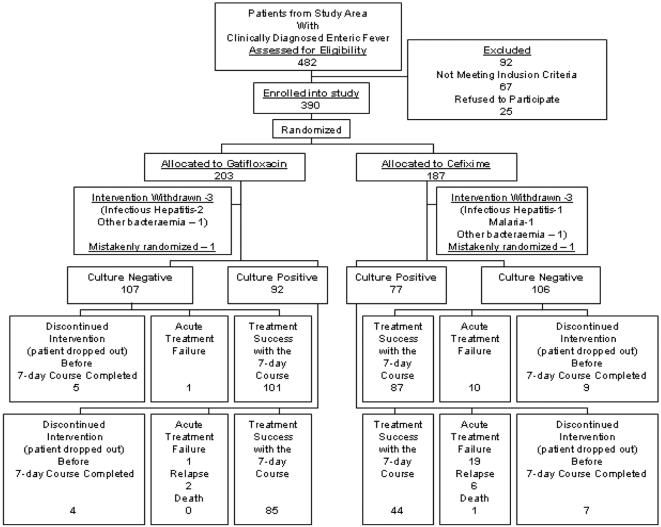
Profile of the Trial. The consort flow diagram showing the flow of participants through the trial.

Of the 482 patients from the study area who were clinically diagnosed with enteric fever, 390 patients were enrolled into the study and randomized. 92 patients were ineligible, the main reason (49 patients) being a history of already having taken antibiotics (fluoroquinolone, macrolide, or third generation cephalosporin) within one week prior to study entry (Figure 1).

Among all randomized patients, 187 patients were assigned to receive cefixime and 203 to gatifloxacin. 77 patients assigned to receive cefixime were blood culture positive for enteric fever whilst 92 of those assigned to receive gatifloxacin were culture positive .There were unequal number of positive patients in each of the study arms. One possible reason for the difference in number of culture positive patients between study arms is that cultures were drawn and culture results obtained after randomization had been done.

### Recruitment

We enrolled patients who presented to the outpatient or emergency department of Patan Hospital, Lalitpur, Nepal from June 5, 2005 to September 8, 2005. All enrolled patients were followed up for at least 10 days after recruitment. Patients with a positive pretreatment blood culture were followed up for 6 months after enrollment.

At the point that the DSMC asked to examine the trial data for the primary outcome measure in positive pre-treatment patients, the median fever clearance time was 92 hours (95% CI, 84–114 hours) for the gatifloxacin treated patients and 138 hours (95% CI, 105–164 hours) for cefixime treated patients. The difference between the two treatment arms was 46 hours (p<0.0001).

### Baseline data

Admission characteristics are shown for all ITT patients (Table 1) and for all PP patients (Table 2). The median age of patients enrolled into the trial was 17 with a range of 2–64 years. There were no baseline differences in the culture positive and culture negative groups, other than temperature at presentation, AST and ALT which were higher and platelets and total WBC which were lower in the culture positive patients as compared to the culture-negative patients. Among all PP patients, there were no differences in the baseline characteristics between the two treatment groups. There were 40 patients, 15 in the gatifloxacin arm and 25 in the cefixime arm, who had taken amoxycillin up to the week before study entry. Of these 4 and 7 were culture-positive respectively.

**Table 1:** Baseline characteristics all patients.

PATIENT CHARACTERISTICS	Culture negative (213)	Culture positive (169)
No of males/No of females	136/77	111/58
Age (yrs)	18 (2–64)	17 (2.75–50)
Number Aged <14 years (%)	79(37.1)	60 (35.5)
Weight (Kg)	44 (10–80)	46 (10–73)
Duration of fever before treatment (days)	5 (0–21)	5 (2–23)
Median oral temperature at presentation(95% CI, range) (in degrees C)	38.7 (38.6–39; 36.5–40.7)	39(38.8–39.2; 36.8–41)
Headache, Number with (%) (median duration [days])	204 (95.7) (4)	164 (97.0) (5.)
Anorexia, Number with (%) (median duration [days])	160 (75.1) [4]	129 (76.3) [4]
Abdominal Pain, Number with (%) (median duration [days])	88 (41.3) [4]	80 (47.3) [4]
Cough, Number with (%) (median duration [days])	83 (39.0) [3]	59 (34.9) [3]
Diarrhoea, Number with (%) (median duration [days])	45 (21.1) [3]	41 (24.3) [3]
Vomiting, Number with (%) (median duration [days])	30 (14.1)[1]	27 (16.0) [2]
Abdominal tenderness ( n [%]) )	32 [15.1]	23 [13.6]
Splenomegaly ( n [%])	18 [8.5]	18 [10.6]
Hepatomegaly ( n [%])	12 [5.76]	9[5.3]
Hematocrit (in%)	40 (27–53)	40 (29–50)
White Cell Count (in ×1000 per microlitre)	7.2 (2.3–24.2)	6.7 (3.0–20.0)
Platelet Count (in ×1000 per microlitre)	192 (66–546)	180 (65–380)
* ALT ( in U/L )	30(11–240)	37 (12–200)
**AST ( in U/L )	43 (20–354)	52 (21–169)
Total Bilirubin ( in mg/dL )	0.8 (0.17–3.6)	0.89 (0.18–3.2)

Baseline epidemiological, clinical and laboratory features at presentation of all intention to treat patients showing a comparison between culture positive and culture negative groups.

* ALT (serum alanine aminotransferase) normal range 5–34 U/L

** AST (serum aspartate aminotransferase) normal range 5–34 U/L

All data presented as median (range) unless specified.

**Table 2:** Baseline characteristics at presentation of culture positive patients.

PATIENT CHARACTERISTICS	GATIFLOXACIN (n = 92)	CEFIXIME (n = 77)
No of males/No of females	67/25	44/33
Age (yrs)	18 (2.75–45)	15 (3–50)
Number Aged <14 years (%)	27 (29%)	33 (43%)
Weight (Kg)	49 (10–70)	42 (11–73)
Duration of fever before treatment (days)	5.2	5.4
Median oral temperature at presentation(95% CI, range) (in degrees C)	39 (38.9–39.2; 37.5–41.0)	39 (38.8–39.2; 36.8–40.5)
Headache, Number with (%) (median duration [days])	88 (95.7%) (5)	76 (98.7%) (4.5)
Anorexia, Number with (%) (median duration [days])	73 (79.3%) (4)	56 (73%) (4)
Abdominal Pain, Number with (%) (median duration [days])	43 (46.7%) (4)	40 (52%) (4)
Cough, Number with (%) (median duration [days])	37 (40.2%) (3)	22 (29%) (3)
Diarrhoea, Number with (%) (median duration [days])	21 (22.8%) (3)	20 (26%) (3)
Vomiting, Number with (%) (median duration [days])	17 (18.5%) (2)	10 (13%) (1.5)
Abdominal tenderness ( n [%]) )	14 (15.2%)	8 (10.4%)
Splenomegaly ( n [%])	10 (10.9%)	8 (10.4%)
Hepatomegaly ( n [%])	5 (5.4%)	4 (5%)
Hematocrit (in%)	41 (30–50)	40 (29–50)
White Cell Count (in ×1000 per microlitre)	6.8(3.0–18)	6.7 (3.1–20)
Platelet Count (in ×1000 per microlitre)	180(65–367)	186 (120–380)
* ALT ( in U/L )	36 (12–180)	39(18–200)
**AST ( in U/L )	53 (24–155)	49 (21–169)
Total Bilirubin ( in mg/dL )	0.85 (0.18–3.2)	0.9 (0.35–2.3)
Positive pretreatment fecal cultures ( n [%])	9 (9.8%)	3 (3.8%)

Baseline epidemiological, clinical and laboratory features at presentation of all blood culture positive patients showing a comparison between the gatifloxacin and cefixime arms.

* ALT (serum alanine aminotransferase) normal range 5–34 U/L

** AST (serum aspartate aminotransferase) normal range 5–34 U/L

All data presented as median ( range) unless specified.

### Numbers analyzed

Analysis was done in all 390 randomised patients (ITT) and separately in 169 patients with positive pre-treatment culture (PP). All endpoints were analysed in the ITT and PP populations, apart from relapse which was only analysed in the PP population.

### Outcomes and estimation

#### Primary outcome

**Figure 2:**
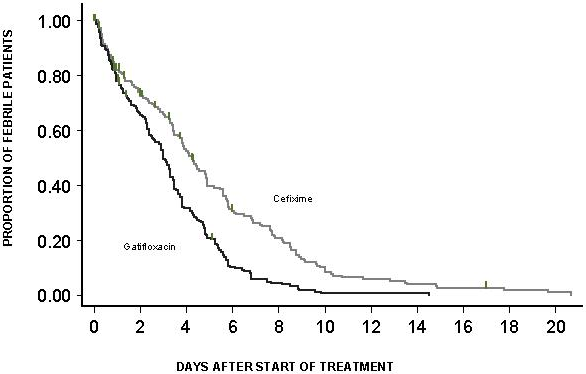
Proportion of all patients still febrile. Kaplan-Meier survival curve showing the proportion of all patients(ITT) still febrile through time.

In all ITT patients, median (95% confidence interval) fever clearance time was 102 (90–117) hours for the cefixime group and 72 (62–80) hours for the gatifloxacin group, logrank test p<0.0001, Hazard Ratio[95%Confidence Interval] = 1.821 [1.466–2.263]. The proportion of all patients failing through time to clear fever is shown in Figure 2. At day 7 fever clearance rate was 73.9% (67.0% – 80.3 %) in cefixime group and 94.2% (90.2% – 96.9%) in gatifloxacin group.

**Figure 3:**
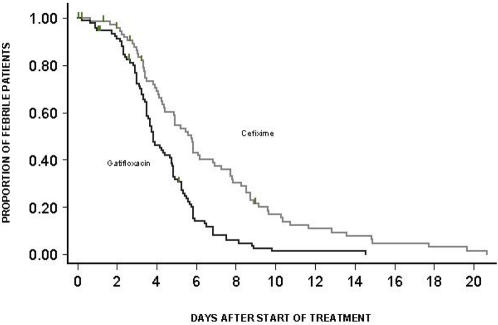
Proportion of culture positive patients still febrile. Kaplan-Meier survival curve showing the proportion of culture positive(PP) patients still febrile through time.

In the PP group, median (95% CI) fever clearance time was 92 hours (84–114 hours) for gatifloxacin recipients and 138 hours (105–164 hours) for cefixime-treated patients (HR[95%CI] = 2.171 [1.545–3.051], p<0.0001). The proportion failing to clear fever for each study drug through time after treatment is shown (Figure 3). At day 7 the fever clearance rate was 62.7% (95 % CI = 51.5%–73.8%) in the cefixime group and 91.8% (95 % CI = 84.8%–96.4%) in the gatifloxacin group.

In the group with negative blood culture but clinically diagnosed enteric fever (Fig 1), the FCT was 82 hours (95% CI = 44–94 hours) for the cefixime group and 39 hours (95%CI = 28–54 hours) for the gatifloxacin group (HR[95%CI] = 1.740 [1.309–2.312], p<0.0001 logrank test).

#### Secondary Outcomes

In the ITT group, overall, 30 out of 167 (18%) in the cefixime group and 2 out of 190 (1%) in the gatifloxacin group were acute clinical failures, OR[95%CI] = 0.049 [0.011–0.207], p<0.001, Fisher's exact test.

In the PP group, 19 out of 70 (27%) patients who completed the 7-day trial had acute clinical failure in the cefixime recipients as compared to 1 out of 88 (1%) in the gatifloxacin recipients (Odds Ratio [95%CI] = 0.031 [0.004 – 0.237], p<0.001). Considering all patients to be failures who dropped out of the study before completion of the seven day treatment course, 26 out of 77 (34%) failed in the cefixime group as compared to 5 out of 92 (5%) in the gatifloxacin group (OR[95%CI] = 0.112 [0.041 – 0.312], p<0.001).

**Figure 4:**
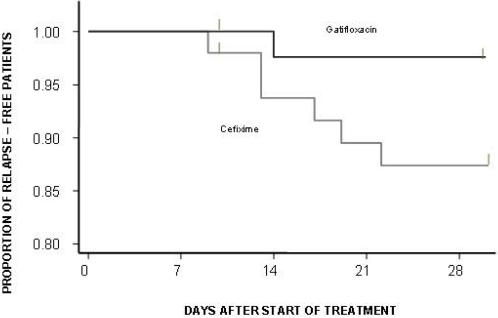
Proportion of relapse free patients. Kaplan-Meier survival curve showing the proportion of relapse free patients in the culture positive population.

138 patients were evaluable for relapse; 20 had acute treatment failure and 11 withdrew from the study before day 7. In total, eight relapses (Figure 1) were observed. Relapse rates were 12.4% (6/51) in the cefixime group and 3.4% (2/87) in gatifloxacin group (HR[95%CI] = 0.185 [0.037–0.915], p =  0.0199). The Kaplan-Meier plots for the time of relapse are shown in Figure 4.

**Figure 5:**
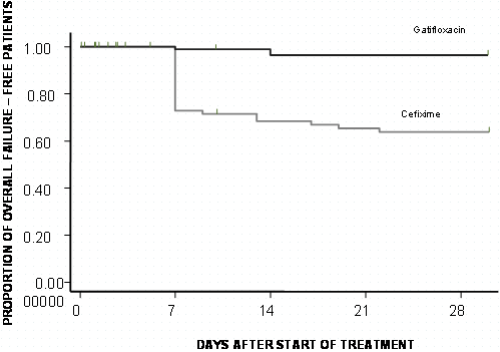
Proportion of overall failure free patients. Kaplan-Meier survival curve showing the proportion of overall failure free patients in the culture positive population.

Overall failures (acute treatment failure plus relapse plus death) were 29 in number (Figure 1). Overall failure rate at 1 month was estimated as 37.6% (95% CI = 27.14% – 50.2%) in the cefixime group and 3.5% ( 95% CI  = 2.2%–11.5%) in the Gatifloxacin group (HR[95%CI] = 0.084[ 0.025–0.280], p<0.0001) ( Figure 5).

From patients with negative cultures, 11 had acute clinical failures, 10 (out of 97, 10%) in Cefixime group and 1 (out of 103, 1%) in the Gatifloxacin group, OR[95%CI] = 0.086 [0.011–0.686], p = 0.004, Fisher's exact test.

Similarly, treating drop-out as treatment failures, we had 50 out of 187 (27%) in the Cefixime group and 15 out of 203 (7%) in the Gatifloxacin group acute treatment failures, OR[95%CI] = 0.219 [0.118–0.405], p<0.001, Fisher's exact test.

### Ancillary analyses

Among all culture positive patients in the cefixime group, one patient (1/70, 1%) had *S.* Paratyphi A cultured from her blood on day 10,but there were no (0/88, 0%) positive blood culture growths in the gatifloxacin group on day 10.

No patient was found to be a persistent carrier of *S.* Typhi or Paratyphi A in their stool. A positive stool culture for *S.* Typhi was seen for one patient on day 10 and for another on day 30. Subsequent cultures were negative for both patients. We were able to obtain stool cultures from 147 (87%), 141 (83%), and 130 (77%) pretreatment blood culture positive patients at one, three, and six months respectively.

#### Microbiology

Antibiotic sensitivity testing revealed that all strains were sensitive to gatifloxacin, cefixime, ceftriaxone or gemifloxacin. One strain was resistant to chloramphenicol, and 136 (83%) of the pretreatment isolates were nalidixic acid resistant strains (NARST). Minimum inhibitory concentration (MIC) was determined for 161 of the pretreatment blood culture isolates. The median (range) MICs for each antibiotic were as follows: gatifloxacin 0.125 (0.006–0.5) µg/mL, cefixime 0.380 (0.016–2.0) µg/mL, nalidixic acid >256 (1.5->256) µg/mL, chloramphenicol 8.0 (1.5->256) µg/mL, ceftriaxone 0.125 (0.047–0.5) µg/mL and gemifloxacin 0.125 (0.004–0.5) µg/mL.

### Adverse events

Among all patients who received cefixime, there was one death, which might have been due to the development of disease-related complications during treatment. This patient was enrolled on the fourteenth day of his illness. On day 6 of treatment the patient complained of reddish stool and petechiae and was immediately admitted to hospital where he developed severe thrombocytopenia and gastrointestinal bleeding. He developed acute respiratory distress syndrome and was mechanically ventilated. He developed disseminated intravascular coagulation and succumbed to his illness on day 21 of entry into the trial. His pretreatment blood culture grew *S.* Paratyphi A which was sensitive to cefixime with an MIC of 0.38 µg/mL. One patient developed erythematous skin rash which needed two doses of oral antihistamine.

Among all patients who received gatifloxacin there were 2 patients with excessive vomiting, which required intravenous antiemetics and fluids and observation in the hospital emergency room for upto 6 hours. There were an additional 23 patients who complained of excessive nausea and occasional vomiting after ingestion of the drug. Of these, two needed oral antiemetics; in the remaining 21 patients no intervention was required.

## Discussion

### Interpretation

In this study examining fever clearance time, acute treatment failure and relapse as indicators of treatment efficacy, that the results raise doubts on the usefulness of cefixime and suggest that gatifloxacin is a potent choice for the treatment of uncomplicated enteric fever.

Febrile illness is one of the most common reasons for presentation to hospitals in many developing countries. In patients with fever, a very common clinical diagnosis is enteric fever, and *S. enterica* serotype Typhi or Paratyphi A are the two most commonly isolated pathogens from the blood in febrile patients in our hospital [15]. Before the advent of multi-drug-resistant (MDR) *S.* Typhi, chloramphenicol, ampicillin or cotrimoxazole were successfully used as the first line drug in the treatment of enteric fever. After the emergence of MDR strains, fluoroquinolones and third-generation cephalosporins have been suggested and used as alternative antimicrobials [16], [13]. However the emergence and spread of point mutations in the *gyrA* gene of the bacterial genome [17] has conferred resistance to nalidixic acid and reduced susceptibility to the commonly used fluoroquinolones such as ofloxacin, leading to a poorer clinical response [18], [19]. A recent study in Viet Nam (CM Parry, unpublished) showed ofloxacin at the dose of 20 mg/kg/day was able to achieve a cure rate in only 64% of patients. In our context of high nalidixic acid resistance, gatifloxacin is the most effective and appropriate choice for treatment of enteric fever. Gatifloxacin (Sandoz, India) is relatively inexpensive (US$1.2 for a 7 day treatment course) and needs to be administered just once a day; both of these features are attractive in this setting. Gatifloxacin has a different binding motif than some other fluroquinolones[20], and this characteristic enables it to retain activity against *Salmonella enterica* serovar Typhi or *Salmonella enterica* serovar Paratyphi A even in the presence of marked reduction in sensitivity to the older fluoroquinolones [17].

Cefixime, a third generation cephalosporin, is widely trusted to be effective for enteric fever as first line treatment, and is also used as second line therapy when initial treatment with a fluoroquinolone in a patient suspected to be enteric fever fails [13]. The fact that we saw a high overall failure rate associated with cefixime despite all of the strains being fully sensitive in vitro to the drug shows that the mechanism of action of cefixime [21], [22] may not be suited to the eradication of *S.* Typhi or Paratyphi A from the body or blood, and the poor intracellular penetration into macrophages and reticulo endothelial tissues where the typhoid organisms colonize [16] may be the cause of high failure rates.

This study was unique in that we used CMAs to simulate a hospital setup in the community. CMA's directly observed patients taking the therapies, monitored fever and identified complications early; these characteristics have not been used in the past for typhoid trials although enteric fever in endemic areas [1] is treated on an outpatient basis. A major advantage of follow-up using CMAs was that the health workers knew the exact house location of the patients, and therefore follow up even after the successful completion of the initial seven-day drug trial was possible. In developing countries follow up of patients can be very difficult because of a lack of a proper address and relative unavailability of other means of communication, for example,a telephone.

In addition to its relevance to culture confirmed enteric fever, another major strength of this large randomized study is that gatifloxacin proved to be more efficacious than cefixime with respect to fever clearance time and failure rates, even in the subgroup of patients who were clinically presumed to have enteric fever but who had a negative blood culture. Antibiotic treatment for typhoid in highly endemic areas is usually started based on the presence of a “syndromic” illness (acute fever for a few days and constitutional symptoms with no known source of infection) before culture results are known. Enteric fever, which continues to be a neglected disease [23], is an important cause of morbidity and mortality, and facilities for blood culture or other reliable methods of diagnosis rarely exist in this setting.

### Generalizability

Despite widespread resistance to Nalidixic acid in Kathmandu, and rising MICs to the older fluoroquinolones, ciprofloxacin and ofloxacin, gatifloxacin has proven to be a potent drug for the treatment of enteric fever. Our study has relevance to South Asia, as resistance to nalidixic acid is widely prevalent there. Inevitably there will be emergence of resistance to gatifloxacin in areas with both MDR and NARST; and in this situation alternative antibiotics like azithromycin may need to be used. Of interest, in keeping with anecdotal reports from elsewhere in South Asia, only one strain was resistant to chloramphenicol in the present study. In areas of the world where chloramphenicol susceptibility has reemerged there may be an argument for reassessing chloramphenicol.

In the present study Gatifloxacin was associated with nausea in 12% of patients and it may be important to forewarn patients of this possible side effect. There have been sporadic reports of dysglycemia caused by gatifloxacin [24]–[26], and a recent population-based, case controlled study examining gatifloxacin usage amongst elderly individuals in Canada (mean age 77 years) who developed dysglycemia [27] also raises possible concerns. We did not do any blood sugar testing to look for dysglycemia. However in a study involving a younger age group where blood sugar testing was done the results revealed no dysglycemia: 887 children were treated with gatifloxacin (10 mg/kg) for otitis media and were followed for a year with no signs of alteration of glucose homeostasis either acutely or otherwise [28]. Clearly, it would be prudent to treat diabetics and elderly people suffering from enteric fever with an alternative antibiotic such as azithromycin and avoid the potential problems in this specific population with gatifloxacin.

#### Limitations of the study

The DSMC advised the Principal Investigators in this study to stop recruitment to the trial based on a priori defined difference (p<0.01) between the two treatment arms in the primary endpoints of the study. It is possible that if the trial had been continued with a larger sample size, other important information could have been gathered. In addition if patients and/or investigators had been blinded to treatment assignments, the study would have been further strengthened; however as in most typhoid trials, it was not possible to do this due to the difference in dosing schedule for the two drugs being compared. Another limitation of this study was that temperature was only measured every 12 hours. However, to address this limitation, and to avoid missing increases in temperature, we checked temperatures for 10 days after enrollment, or for 48 hours after resolution of fever, whichever came later, in all patients. Finally, a telephone or internet based system of randomization would be ideal, but such a system does not exist here.

### Overall evidence

We have compared the outcomes from our trial with those of other comparable studies, identified from a recent Cochrane review [4], WHO typhoid guidelines [10], and a search of Medline using these terms: cefixime, typhoid trials. The findings in our study are consistent with those of a 1995 study done in Viet Nam which showed that cefixime (20 mg/kg/day) for 7 days was inferior to ofloxacin (10 mg/kg/day) for 5 days in the treatment of MDR typhoid fever in children[14]. However other studies have suggested cefixime can be successful in the treatment of enteric fever [29]–[33]. Overall these studies, both descriptive and randomized, have examining the use of cefixime in confirmed enteric fever (total of 292 patients) and with treatment durations of mostly 14 days, have found failure rates ranging from 4% to 23%. Besides the general undesirability of a longer course with cefixime with increased morbidity and possibly complications, this drug is also more expensive (a 7-day course costs US $7 (Blue Cross Laboratories, India)). The present study is the largest randomized controlled trial ever conducted with cefixime in enteric fever and clearly shows, even in a setting with fully sensitive strains, that cefixime is a poor drug for this disease. These findings are contrary to the recommendation by many sources[13], [1] including the World Health Organization[10] that cefixime can be used as first or second line therapy in the treatment of enteric fever. Based on the present study, we believe gatifloxacin to be an optimal choice in the treatment of uncomplicated enteric fever.

## Supporting Information

Checklist S1 CONSORT Checklist
(0.05 MB DOC)

Protocol S1 Trial Protocol
(0.05 MB DOC)
