# Supplementary material for: An Open Randomized Comparison of Gatifloxacin versus Cefixime for the Treatment of Uncomplicated Enteric Fever
Source: PLoS One. 2007 Jun 27;2(6):e542. doi: 10.1371/journal.pone.0000542 (PMC1891439; doi:10.1371/journal.pone.0000542)
Supplement: Protocol S1 — Trial Protocol (0.05 MB DOC) [file pone.0000542.s002.doc]

#### Aim

A single open randomized comparison of Gatifloxacin and Cefixime for 7 days in patients with uncomplicated typhoid fever.

**Patient selection:**

Patients will be eligible to be admitted to the study if they have suspected enteric fever provided they come to Patan Hospital outpatient or ER from Lalitpur area in Kathmandu Nepal. The home site must be accessible to our Community Medical Assistant (CMA) who will monitor the temperature 12 hourly and provide directly observed therapy. Patients will be eligible if:

1. They give fully informed consent.
2. They are not obtunded or shocked, are not visibly jaundiced and have no signs of gastrointestinal bleeding or any other evidence of severity.
3. No previous history of hypersensitivity to either of the trial drugs
4. No known previous treatment with a quinolone antibiotic or 3rd generation cephalosporin or macrolide within one week of hospital admission. (Patients who have received chloramphenicol, ampicillin, or co-trimoxazole will be included as long as they have not shown evidence of clinical response).
5. They are not pregnant.
6. They do not have a history of seizures.

#### Treatment

This will be an open, randomised comparison. The treatment allocation will be in an opaque envelope. Once the envelope is opened the patient will be considered to have entered the trial whatever happens subsequently. Patients will be randomized to group A or group B.

Group A: Gatifloxacin 10mg/kg/day for 7 days

GroupB: Cefixime 20mg/kg/day for 7 days

#### Observations and Investigations

a) Prior to admission to the study

1. Full history and clinical examination.
2. Chest X-ray and other radiological investigations, including abdominal ultrasound, as clinically indicated.
3. Hct, white cell count/differential, platelet count, SGOT, SGPT, CRP and urinalysis
4. Microbiology:

- Blood cultures (5-8 ml)
- Stool culture pre-treatment

# **Day 1 - Discharge (Patients will be managed as outpatients and seen at home every day)**

- Oral temperature will be recorded at 12 hourly intervals and a daily symptom and activity record will be kept. The study drug will be given under direct observation. The CMAs and study physicians will meet twice daily to discuss patients under their charge.

## **Day 10**

- Hct, white cell count, platelets, SGOT, SGPT will be repeated as indicated by results and clinical condition.
- Blood cultures will be performed on day10.
- Faeces will be re-cultured on day 10.
- Abnormal radiological investigations will be repeated as appropriate.
- All isolates from the initial illness, “relapse” and asymptomatic carriage on follow-up will be stored at - 20^0^C for antibiotic susceptibility testing, plasmid typing and pulse field gel electrophoresis to distinguish relapse and re-infection.

**Out-patient follow up** - One month

- Full history and relevant examination will be performed.
- Any previously abnormal laboratory investigation will be repeated. Stool culture x 1 will be mandatory.
- A full set of microbiological cultures (blood) will only be performed if symptoms and signs suggest further infection.
- Patients will be visited at their home to ask about clinical symptoms and obtain at least one stool specimen.

**Out-patient follow up** – Three and six months

- Full history and relevant examination will be performed.
- Any previously abnormal laboratory investigation will be repeated. Stool culture x 1 will be mandatory.
- A full set of microbiological cultures (blood, urine) will only be performed if symptoms and signs suggest further infection.
- Patients will be visited at their home to ask about clinical symptoms and obtain at least one stool specimen.

#### Treatment outcome assessment

The response to treatment will be assessed by

- FEVER CLEARANCE TIME(Time to first fall ≤ 37.5^0^C,oral, and to remain ≤ 37.5^0^C for 48 hours)
- TREATMENT FAILURES (see below)
- CLINICAL PARAMETERS

(resolution of clinical symptoms and signs; requirement for admission)

- Eradication of bacteraemia at Day 10
- Time to eradication of stool carriage
- Development of complications
- Need for retreatment
- Occurrence of relapse of infection at follow up

#### Failure of treatment

Treatment failure will be defined as

- the persistence of fever( >38 C) [see the table below: management of clinical failures]
- the development of any severe complication.
- A relapsed patient ( within one month of treatment with positive blood culture)

Patients who fail gatifloxacin will be treated with ceftriaxone 40mg/kg/day for 14 days and patients who fail cefixime will be treated with olfloxacin 20mg/kg/ day for 14 days.

#### Removal from the study

Patients who fail treatment or have organisms other than S.typhi or S.paratyphi A grown from admission blood will be removed from the study and given appropriate treatment, but their outcome will be recorded.

#### Adverse events

All adverse events will be fully recorded including duration, severity, outcome and relationship to study drug.

**Out-patient follow up (4-6 weeks post treatment)**

1. Full history and relevant examination will be performed. Any previously abnormal laboratory investigation will be repeated, stool culture
2. A full set of microbiological cultures (blood,) will only be performed if symptoms and signs suggest further infection.
3. Patients with convalescent stool carriage of S.typhi or S.paratyphi A will have arrangement will be made for further follow up. An ultrasound will be performed to exclude biliary or kidney stones if carriage is persistent.
4. If carriage is present following treatment a further course of treatment will be given according to the antibiotic sensitivity of the isolate.

**Management of Clinical Failures – DM Study**

Definitions:

**Clinical Success** is sustained resolution of fever – ie temperature ≤ 37.5 for at least 24 hours

**Fever Clearance Time** – Time from first temperature recording at trial entry to the first temperature recording ≤ 37.5 C (sustained defervescence, ie temperature always ≤ 37.5C).

| **Temperature Day 7** | **Treatment Arm** | **Observation** | **Action** | **Temperature Day 10** | **Observation** | **Action** |
| --- | --- | --- | --- | --- | --- | --- |
| Temperature  ≤ 37.5 | Gatifloxacin  Cefixime | Clinical Success | No action, follow up on days 8 - 10 |  |  |  |
| Temperature >37.5 , <38C,  Patient improving | Gatifloxacin | Possible success | Observe days 8 - 10 | ≤ 37.5 | Clinical Success | No Action |
|  |  |  |  | >37.5 | Clinical Failure | Retreat with Ceftriaxone |
| Temperature >37.5 , <38C  Patient improving | Cefixime | Possible success | Observe days 8 - 10 | ≤ 37.5 | Clinical Success | No Action |
|  |  |  |  | >37.5 | Clinical Failure | Retreat with Ofloxacin |
| Temperature ≥38C | Gatifloxacin | Clinical Failure | Retreat with Ceftriaxone 14 days |  |  |  |
| Temperature ≥38C | Cefixime | Clinical Failure | Continue Cefixime up to and including day 10. | >37.5 C | Clinical Failure | Retreat with Ofloxacin |

Any Microbiological Failure (ie positive blood culture on day 10) requires retreatment with Ceftriaxone (for Gatifloxacin patients) or Ofloxacin (for Cefixime patients). Retreatment is to be guided by the antibiotic sensitivities of the infecting organism if known. Note that antibiotic resistance does not appear to arise *de novo* during patient treatment, so the susceptibility pattern from the day 1 blood culture can be used if available.

| Centigrade | Fahrenheit | Centigrade | Fahrenheit |
| --- | --- | --- | --- |
| 37.5 | 99.5 | 38.5 | 101.3 |
| 38 | 100.4 | 39 | 102.2 |
